# Integrated Whole-Cell Biocatalysis for Trehalose Production from Maltose Using Permeabilized *Pseudomonas monteilii* Cells and Bioremoval of Byproduct

**DOI:** 10.4014/jmb.2202.02028

**Published:** 2022-05-20

**Authors:** Srisakul Trakarnpaiboon, Verawat Champreda

**Affiliations:** Enzyme Technology Research Team, Biorefinery and Bioproduct Technology Research Group, National Center for Genetic Engineering and Biotechnology, 113 Thailand Science Park, Paholyothin Rd., Klong Luang District, Pathumthani 12120, Thailand

**Keywords:** Trehalose, trehalose synthase, permeabilized cell, maltose, *Pseudomonas monteilii*

## Abstract

Trehalose is a non-conventional sugar with potent applications in the food, healthcare and biopharma industries. In this study, trehalose was synthesized from maltose using whole-cell *Pseudomonas monteilii* TBRC 1196 producing trehalose synthase (TreS) as the biocatalyst. The reaction condition was optimized using 1% Triton X-100 permeabilized cells. According to our central composite design (CCD) experiment, the optimal process was achieved at 35°C and pH 8.0 for 24 h, resulting in the maximum trehalose yield of 51.60 g/g after 12 h using an initial cell loading of 94 g/l. Scale-up production in a lab-scale bioreactor led to the final trehalose concentration of 51.91 g/l with a yield of 51.60 g/g and productivity of 4.37 g/l/h together with 8.24 g/l glucose as a byproduct. A one-pot process integrating trehalose production and byproduct bioremoval showed 53.35% trehalose yield from 107.4 g/l after 15 h by permeabilized *P. moteilii* cells. The residual maltose and glucose were subsequently removed by *Saccharomyces cerevisiae* TBRC 12153, resulting in trehalose recovery of 99.23% with 24.85 g/l ethanol obtained as a co-product. The present work provides an integrated alternative process for trehalose production from maltose syrup in bio-industry.

## Introduction

Trehalose (also known as α-D-glucopyranosyl-α-D-glucopyranoside) is a non-reducing disaccharide composed of two glucose molecules linked through an α,α-(1,1)-glycosidic bond [1]. It is widely used in food, cosmetic, and medicinal products, in which its functions range from a sweetener to a biomaterial stabilizer. Trehalose was approved as a food ingredient and received generally recognized as safe (GRAS) status in 2000 [2]. At present, the development of new technologies for trehalose production is of great interest as the demand is growing [3].

Currently, trehalose can be produced by two main enzymatic processes. In the first, maltooligosyltrehalose synthase (MTSase, E.C. 5.4.99.15) and maltooligosyltrehalose trehalohydrolase (MTHase, E.C. 3.2.1.141) are applied for production of trehalose from starch or maltodextrins. However, the MTSase/MTHase process involves two complicated enzymatic steps and generates branched starch, maltose, and maltotriose as byproducts, which limits the efficiency of trehalose production [4]. Alternatively, trehalose synthase (TreS, E.C. 5.4.99.16) is used to produce trehalose from maltose. This one-step process, through intermolecular rearrangement mechanism, is considered advantageous for its simplicity and cost effectiveness [5]. Most studies on trehalose production by TreS have focused on the use of recombinant enzymes, and several TreS genes have been cloned from different bacterial strains and heterologously expressed in *Escherichia coli* [4]. However, the process economics depend on the cost of recombinant enzyme production, which is usually relatively high, making this approach economically unattractive [6-8].

Whole-cell catalysis is a simple process that offers high stability and biocatalyst resistance to environmental perturbations compared to free enzymes. Since the process does not require enzyme production and isolation, the reactions can be more commercially feasible [9]. Permeabilization of cells with detergents and solvents such as Tween 20, Tween 80, Triton X-100, acetone, chloroform, ethanol, methanol, and toluene have been applied to enhance the transfer of substrates and products across the cell membrane [10]. Permeabilized recombinant *E. coli* cells have been studied for trehalose production [11, 12]. However, the application of native microorganisms is preferred due to strict regulation on the use of recombinant cells in bio-industry to produce ingredients for food and healthcare products [13].

In the present work, *P. monteilii* TBRC 1196, a wild-type strain capable of producing TreS, was used as the whole-cell biocatalyst for trehalose production from maltose [14]. We optimized various factors related to trehalose synthase activity and investigated the cell permeabilizing conditions. The conversion process was optimized by an experimental design approach and demonstrated in a lab-scale bioreactor. We then examined an integrated process for trehalose production and byproduct bioremoval. In addition, a selected strain of *Saccharomyces cerevisiae* was applied to convert the residual maltose and glucose to ethanol in the same bioreactor in order to obtain high-purity trehalose. Our study provides a platform for further developing the whole-cell catalytic process for trehalose production from maltose syrup in bio-industry.

## Materials and Methods

### Microorganism and Culture Conditions

*P. monteilii* TBRC 1196 and *S. cerevisiae* strains were obtained from Thailand Bioresource Research Center (TBRC: www.tbrcnetwork.org). The bacterial strain was maintained on nutrient agar and grown in nutrient broth at 30°C. The yeast strains were maintained on YPD agar and grown in YPD broth at 30°C.

### Factors Affecting Trehalose Synthase Production by *P. monteilii* TBRC 1196

The effects of factors including temperature, inoculum size, shaking speed and maltose concentration on TreS production by *P. monteilii* TBRC 1196 were investigated by varying fermentation conditions. *P. monteilii* TBRC 1196 was pre-cultured at 30°C with rotary shaking at 150 *×*g for 18-20 h. The cells were collected by centrifugation at 4°C and 6,000 *×*g for 5 min. The cell pellets were resuspended in sterile distilled water to adjust the OD_600_ to 5. Five milliliters of cell suspension were transferred into a 250 ml Erlenmeyer flask containing 50 ml of the pre-defined production medium (%w/v, maltose 10%, peptone 5%, yeast extract 1%, K_2_HPO_4_ 1%, NaH_2_PO_4_ 1% and MgSO_4_.7H_2_O 0.5%) and incubated for 24 h. The effect of temperature was studied at 25, 30, 35, 40, and 45°C. The effect of inoculum size was determined at 5, 10, 15, and 20% (v/v) under the optimal culture conditions. The effect of shaking speed on enzyme production was examined at 150, 200, and 250 rpm, while the effect of maltose concentration was studied using 0, 2.5, 5.0, 7.5, and 10% (w/v) under the optimal conditions.

To determine TreS activity, the culture broth was centrifuged at 6,000 *×*g for 5 min at 4°C. Then, the cells were suspended in 1 ml of 0.02 mM phosphate buffer (pH 7.0) and lysed using an Ultrasonic Processor (VCX 130 PB, Sonics & Materials, Inc., Newtown, CT) with a 3 mm diameter probe at a frequency of 20 kHz and 80% amplitude for 30 sec. Then, 0.5 ml of crude cell extracts was mixed with 0.5 ml of maltose solution (2% w/v) in phosphate buffer (pH 7.0). Following incubation of the reaction mixture at 40°C for 3 h, the reaction was stopped by heating at 95°C for 15 min. The mixture was then centrifuged at 4°C and 6,000 *×*g for 5 min. The amount of trehalose in the reaction mixture was measured by using a Megazyme Trehalose Assay Kit (Megazyme International, Ireland). One unit of trehalose synthase activity was considered equal to the amount of enzyme required to produce 1 nmol/min of trehalose from maltose.

### Permeabilization of *P. monteilii* TBRC 1196 Cells

The bacterial strain was pre-cultured in 50 ml nutrient broth medium in 250 ml Erlenmeyer flasks and grown at 30°C for 24 h with shaking at 200 *×*g. The culture (25 ml) was used as an inoculum in 250 ml of the production medium in 1 L Erlenmeyer flasks and further incubated under the same conditions. The cells were pelleted by centrifugation at 6,000 *×*g at 4°C for 10 min. The pelleted cells were resuspended in an appropriate volume of 50 mM sodium phosphate buffer (pH 7.0) to yield the final OD_600_ of 50, which corresponded to a biomass concentration close to 19.5 g/l on a dry weight basis. The concentrated suspension was centrifuged at 6,000 *×*g for 10 min at 4°C and stored at -20°C.

The permeabilized cell preparation method was modified from Shin *et al*. 2016 [15]. The cells were thawed and resuspended in solutions containing different detergents selected from among Tween 20 or Tween 80 (5% v/v), Triton X-100 (0.75% v/v), and ethanol (40% v/v). The bacterial cell suspensions were incubated at 4°C for 15 min before centrifugation and washing twice with 50 mM sodium phosphate buffer (pH 7.0).

### Determination of Triton X-100 Concentration and Incubation Time

To investigate the effect of the Triton X-100 concentration and incubation time, the detergent concentration was varied at 0.25, 0.50, 0.75, and 1% (v/v) with an incubation time of 15, 30, and 45 min. The treated cells were used as the whole-cell biocatalysts for trehalose production, which was studied with 50 g/l of maltose at 40°C and pH 7 for 24 h.

### Optimization of Trehalose Production by Permeabilized Cells Using CCD

Trehalose production by permeabilized cells of *P. monteilii* TBRC 1196 was optimized using CCD with five levels and two variables (pH and temperature) using Design Expert 13 software (STAT-EASE Inc., USA). The trehalose yield (%) was set as the dependent variable. The two factors distributed across four levels and one center medium point are shown in Table 1. The lower and upper limits of the two variables were fixed based on the previous experimental work reported by Cai *et al*. 2018 [4]. Thirteen treatments were established based on CCD with two factors. The trehalose production was performed using 50 g/l maltose as a substrate in a 25 ml Erlenmeyer flask for 24 h. The experiment with CCD was done in triplicate to minimize the experimental error.

The analysis of variance (ANOVA) table was generated and the effect and regression coefficients of individual linear, quadratic, and interaction terms were determined. The significances of all terms in the polynomial were judged statistically according to the *p*-value compared to the significance level of 95%. The coded mathematical model for the quadratic model can be given as Eq. (1):

$$Y=\beta_{0}+\sum_{i=1}^{k}\beta_{i}X_{i}+\sum_{i=1}^{k}\beta_{ii}X_{i}^{2}+\sum_{i}\sum_{j}\beta_{j}X_{i}X_{j}+\varepsilon$$ (1)

where *Y* is the predicted response,

*k* is the number of factor variables,

*β*_0_ is the model constant,

*β*_i_ is the linear coefficient,

*X*_i_ is the factor variable in its coded form,

*β*_ii_ is the quadratic coefficient, and

*β*_ij_ is the interaction coefficient.

### Effect of Substrate and Permeabilized Cell Concentration on Trehalose Conversion

To determine the effect of substrate concentration on trehalose production by permeabilized *P. monteilii* TBRC 1196, the permeabilized cells prepared by 1% (w/v) Triton X-100 treatment were mixed with 50, 100, and 150 g/l of maltose with the total reaction volume of 50 ml in 250 ml Erlenmeyer flasks. The mixtures were incubated in potassium phosphate buffer (pH 8.0) at 35°C for 24 h. The trehalose formation was determined by HPLC.

The effect of permeabilized cell concentration on trehalose conversion was investigated using different initial concentrations of permeabilized cells (62.7, 94.0, 156.7, and 219.3 g/l) with 100 g/l of maltose as the substrate in 50 mM sodium phosphate buffer (pH 8.0). The reactions were incubated at 35°C with shaking at 100 rpm for 24 h. The concentrations of trehalose, maltose and glucose were determined by HPLC.

### Production of Trehalose by Permeabilized Cells in Bioreactor

Permeabilized *P. monteilii* TBRC 1196 cells were prepared according to the optimal permeabilization condition (1% (w/v) Triton X-100, 15 min) using maltose (100 g/l) as the substrate in 50 mM sodium phosphate buffer (pH 8.0). The batch trehalose production process was studied in a 1 L stirred-tank bioreactor (MDET-N-2L, B.E. Marubishi, Pathum Thani, Thailand) using a working volume of 500 ml at 35°C with an agitation speed of 100 rpm. Samples were taken at intervals, and the produced sugars were measured by HPLC.

### Simultaneous Trehalose Purification and Ethanol Production

To obtain a yeast strain that could utilize glucose and/or maltose but not trehalose, 18 strains of *S. cerevisiae* from our laboratory stocks were spotted on YNB agar plates (10 g/l Yeast Extract; 20 g/l Peptone and 15 g/l, Agar) supplemented with 20 g/l glucose, maltose, or trehalose. The plates were incubated at 30°C for 48 h. The selected strains were used for simultaneous trehalose purification and ethanol production. The inoculum was prepared by transferring one loop full of 24 h culture grown on YPD agar into 10 ml YPD broth followed by incubation on a rotary shaker at 200 rpm and 30°C for 20 h. The yeast cells were then pelleted by centrifugation and washed with sterile distilled water. The initial cell concentration was adjusted to an OD_600_ of 1 and inoculated into 10 ml YNB broth supplemented with 20 g/l of glucose or maltose with an initial pH of 6.5 at 200 rpm and 30°C for 24 h. Sugars and ethanol concentrations at 24 h were analyzed by HPLC.

The bioremoval step was carried out in a 1 L shaking flask with 250 ml working volume. The trehalose solution was supplied with nitrogen source selected from (1) 10 g/l yeast extract plus 20 g/l peptone or (2) 1 g/l (NH_4_)_2_SO_4_, plus 1 g/l KH_2_PO_4_ and 0.7 g/l of MgSO_4_·7H_2_O. Strain *S. cerevisiae* TBRC 12153, which is capable of glucose up-take with marginal assimilation of trehalose, was used for removal of the residual glucose and maltose in the product mixture obtained from the synthesis reaction using permeabilized *P. monteilii*. The inoculum was prepared by transferring one loop full of 20-24 h culture grown on YPD agar into 50 ml YPD broth and incubated on a rotary shaker at 30°C and 200 rpm for 24 h. An initial cell concentration of 20 OD_600_ was added into the product mixture. Then, 0.1 % (w/w) of glucoamylase (GA Extra, Siam Victory Chemicals Co., Ltd., Thailand) was added in the solution. Fermentation was conducted with an initial pH of 6.5 at 30°C with shaking at 150 rpm. Samples were taken at 15, 24, and 48 h. The concentrations of glucose, maltose, trehalose and ethanol were analyzed by HPLC.

### Integrated Trehalose Production and Byproduct Bioremoval in Bioreactor

Trehalose conversion by permeabilized *P. monteilii* TBRC 1196 cells was studied in a 1 L stirred-tank bioreactor (model MDET-N-2L, B.E. Marubishi, Thailand) containing 0.5L of 100g/l maltose and 97.53g/l of the permeabilized cells. The reaction was controlled at 35°C and pH 8.0 with an agitation at 100 rpm for 15 h. Then, the temperature and pH were decreased to 30°C and 6.0. The agitation was then increased to 200 rpm, and the *S. cerevisiae* TBRC 12153 culture with an initial cell concentration of 20 OD_600_ was inoculated at 5% into the bioreactor to remove the residual glucose and maltose. Then, 0.1% (w/w) of glucoamylase (GA Extra, Siam Victory Chemicals Co., Ltd.) was added in the mixture and the process was continued under the experimental conditions for 15 h. Samples were analyzed by HPLC to measure the concentrations of glucose, maltose, trehalose and ethanol.

### Analysis

Maltose, trehalose and glucose were measured following the method of Kuschel *et al*. 2017 [16] with minor modifications. The concentrations (%, w/v) of glucose, maltose and trehalose were analyzed by HPLC using an LC-20A Series (Shimadzu-GL Sciences, Japan) equipped with a Shodex HILICpak VG-50 4E column (4.6 × 250 mm, 5 μm) at 40°C. Acetonitrile : ultrapure water (80 : 20 v/v) was used as the mobile phase at a flow rate of 1.0 ml/min. Ethanol present in samples were determined using a Bio-Rad HPX-87H Organic Acid Analysis Column (USA) at 65°C. The mobile phase was 5 mM H_2_SO_4_ at 0.6 ml/min.

### Statistical Methods

The data values of all experiments were reported as the mean ± SD. Significant differences among groups were calculated using one-way ANOVA, followed by multiple comparisons using Duncan’s test (at *p* < 0.05), provided by the statistical software SPSS 11.5 (SPSS Inc., USA). *p*-values less than 0.05 were considered statistically significant and included in the study.

## Results and Discussion

### Effect of Cultivation Factors on TreS Production by *P. monteilii*

The effects of temperature, inoculum size, shaking speed and maltose concentration on TreS production by the bacterial strain were studied. Table 2 showed that the optimal cultivation temperature was 35°C, which yielded the maximum TreS activity of 371.80 ± 7.29 U/ml from a 10% inoculum size. The optimal shaking speed for maximum production of TreS was 200 rpm (442.62 ± 2.53 U/ml). The effect of maltose concentration was studied under the conditions with optimized temperature, inoculum size, and shaking speed. The optimal maltose concentration at 5% (w/v) gave the highest TreS production of 472.19 ± 0.20 U/ml. Increasing trehalose accumulation was reported under stress conditions [17]. This explains the lower TreS activity observed when the shaking speed was further increased above 200 rpm, which led to increased oxygen transfer and lower stress, which were reflected in higher cell growth. This also explains the increasing trend of TreS activity with increasing maltose concentration from 0-5% due to increasing osmotic stress [18, 19].

### Effect of Permeabilizing Agents and Conditions on Trehalose Production

**Fig. 1:**
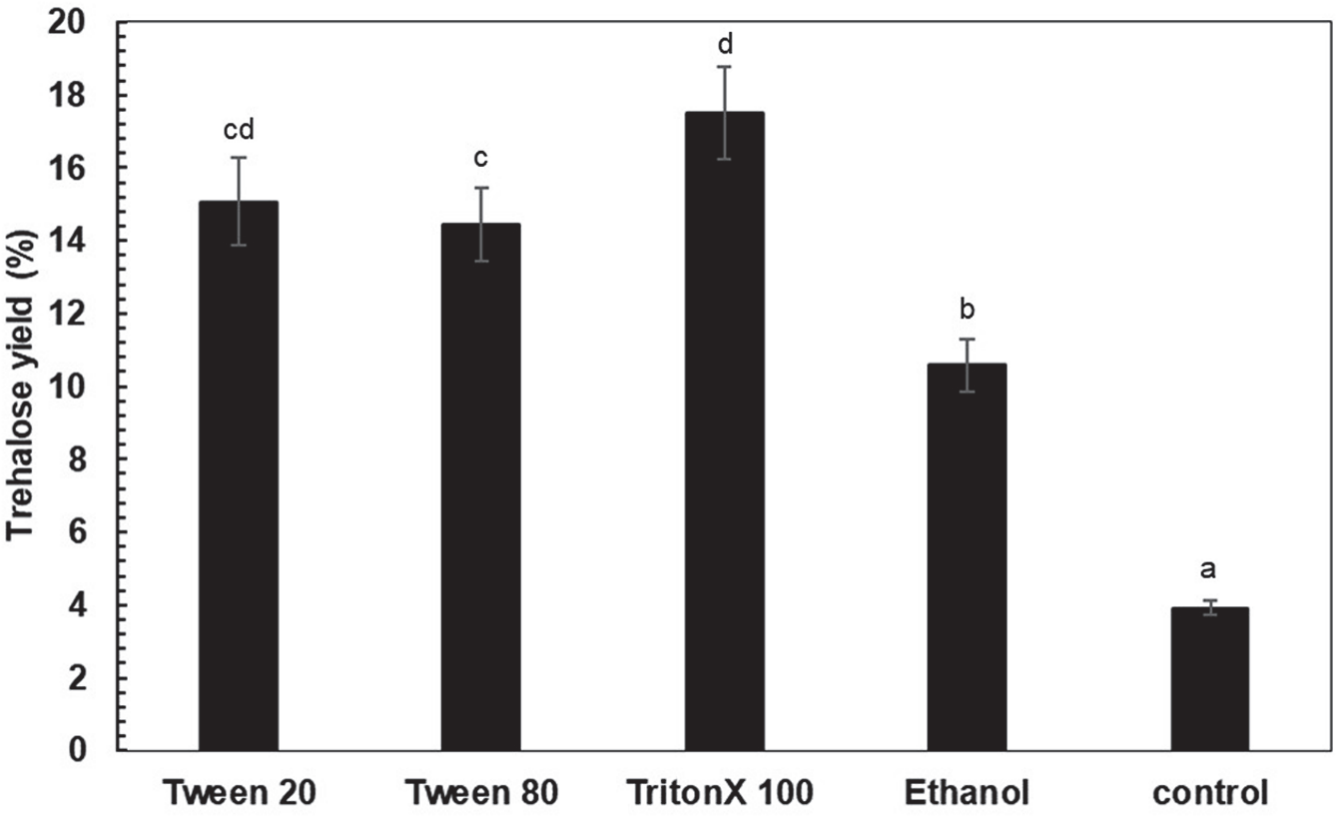
Effects of reagent types on the permeabilization of *P. monteilii* TBRC 1196 cells for trehalose production. The reactions contained 50 g/l of maltose and were incubated at 40°C, pH 7.0 for 24 h. Different letters are significantly different (*p* < 0.05, Duncan’s multiple range test).

The effects of permeabilizing agent types and conditions in the biocatalyst preparation step on trehalose yield were investigated. According to Fig. 1, treatment of *P. monteilii* TBRC 1196 cells with Triton X-100 gave the highest trehalose yield of 17.50% under the experimental conditions. Cell permeabilization with Tween 20, Tween 80 and ethanol gave a lower trehalose yield of 10.57-15.36%.

**Fig. 2:**
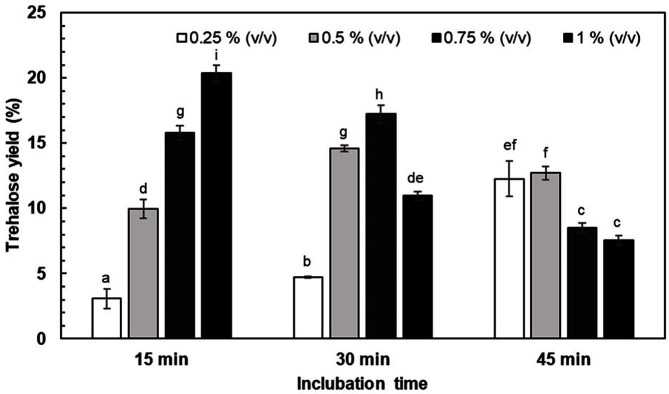
Effects of Triton X-100 concentration on the permeabilization of *P. monteilii* TBRC 1196 cells for trehalose production. The reactions contained 50 g/l of maltose and were incubated at 40°C, pH 7.0 for 24 h. Different letters are significantly different (*p* < 0.05, Duncan’s multiple range test).

To optimize Triton X-100 concentration and incubation time, the cells were treated with different concentrations of the detergent at various incubation times. The highest trehalose yield of 20.34 %, equivalent to 5.19-fold compared to the control using the untreated cells, was obtained using the cells treated with 1% (w/v) Triton X-100 for 15 min (Fig. 2). When the incubation time was longer than 15 min, decreasing trehalose yield was observed. A concentration of Triton X-100 lower than 1% did not increase the trehalose yield compared to the control using the untreated cells.

Many reagents have been applied for permeabilization of various organisms with varying efficiencies [20]. Detergents and solvents such as Tween 20, Tween 80, Triton X-100 and ethanol were widely used for cell permeabilization in biocatalytic processes for production of other sugar products, *e.g.*, D-psicose [9]. Triton X-100 was found to be the most efficient permeabilization agent for *P. monteilii* TBRC 1196 with a lower effective concentration than other reagents. Compared to other reagents, Triton X-100 could disrupt the cell membrane at a relatively low concentration [21]. In previous studies, Triton X-100 at 0.5-2%, w/v was found to be the suitable concentration for permeabilization of several microbial cells, for example, *Corynebacterium glutamicum*, *Waltomyces lipofer* and *Rhodobacter sphaeroides* [15, 22, 23].

### Optimization of Reaction Parameters for Trehalose Production

In the present study, trehalose production by permeabilized *P. monteilii* TBRC 1196 cells was optimized using CCD, based on response surface methodology. The effects of two independent variables are presented in Table 3 along with the mean observed values. The maximum trehalose yield (37.16%) could be achieved at pH 8 and 35°C.

An ANOVA was performed for evaluation of the variables and their possible interactions as shown in Table 4. The second order quadratic model showing the F-value of 5.85 implies that the model is significant. B, AB, A², B² are significant model terms (*p* < 0.05). The value of R^2^ obtained in this study was 0.9868. The values of adj-R^2^ and pred-R^2^ were 0.9774 and 0.9292, respectively, suggesting good fitness of the data. The Lack of Fit F-value of 2.94 implies the Lack of Fit is not significant relative to the pure error. The quadratic equation in Eq. (2) describes the correlation between the variables and the yield of trehalose in coded terms:

$$\text{Y=35.31-0.8069A-2.13B+2.46AB-7}{\text{.41A}}^{\text{2}}\text{-14}{\text{.10B}}^{\text{2}}$$ (2)

where Y is the response (trehalose yield, %) and A and B are the coded forms of the independent factors (pH and temperature).

**Fig. 3:**
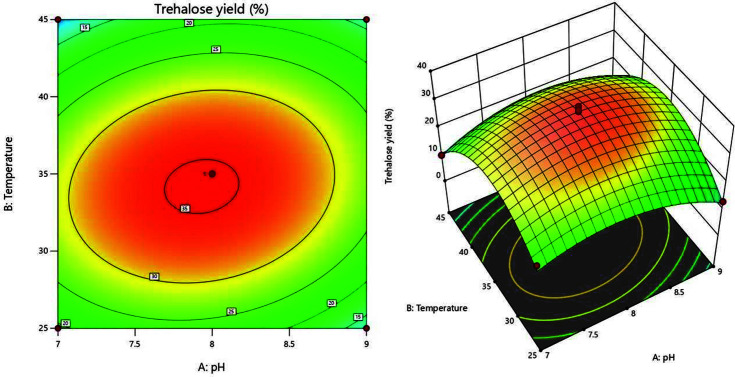
Contour plots showing interactions between independent variables (pH vs. temperature). The reactions contained 50 g/l of maltose and were incubated at different pH and temperatures for 24 h.

The interaction between the response and variables was visualized by a response surface 3D curve, contour plot, and interaction plots constructed according to the quadratic model. Fig. 3 illustrates the interaction between pH and temperature and their effects on trehalose yield in this study. The maximum predicted yield is located in the smallest eclipse of this graph. To verify the optimization results, the percentage errors between actual and predicted values of all experiments are shown in Table 5. The errors were all in an acceptable range (< 30%). The suggested optimal condition was pH 8.0 and 35°C.

### Effect of Substrate Concentration and Cell Loading on Trehalose Production

The effect of the substrate concentration on the yield of trehalose by the permeabilized cells was examined at pH 8.0 and 35°C. The yields of trehalose at the maltose concentrations of 50, 100, and 200 g/l were 45.58, 51.79, and 43.16 g/l, respectively (Table 6).

**Fig. 4:**
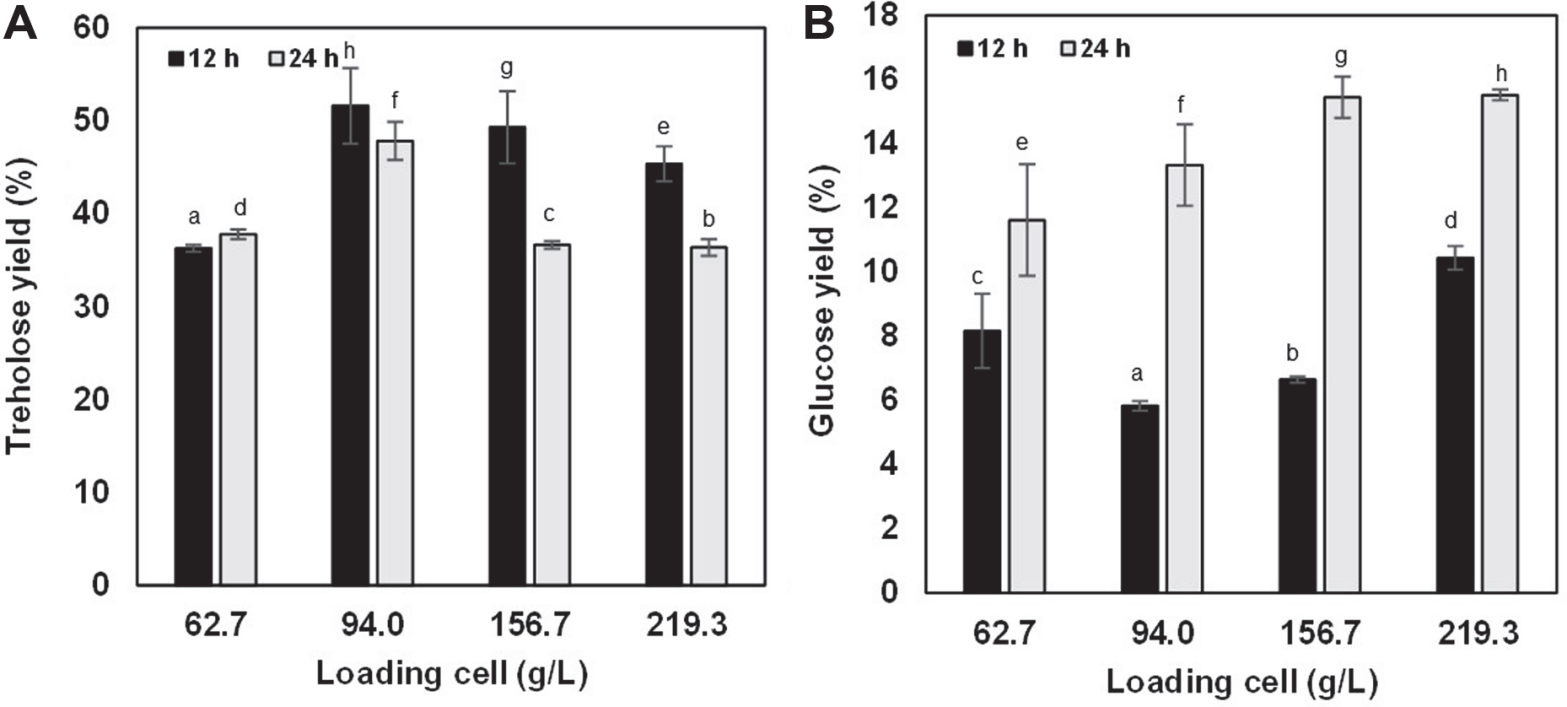
Trehalose (**A**) and Glucose (**B**) yield from maltose conversion by permeabilized *P. monteilii* TBRC 1196 cells. The reactions contained 100 g/l maltose in 50 mM potassium phosphate buffer (pH 8.0) and were incubated at 35°C for 24 h. Different letters are significantly different (*p* < 0.05, Duncan’s multiple range test).

The effect of initial loading of the permeabilized cells on trehalose production was studied. Increasing concentrations of the permeabilized cells from 62.66 to 93.99, 156.65 and 219.31 g/l resulted in improved trehalose yield (Fig. 4A). The optimum concentration of cell loading was 94 g/l with 51.6% yield at 12 h, while further increase in cell loading showed a decline in the trehalose yield (Fig. 4A). Moreover, reduction of the trehalose yield at 24 h was observed. Increase in glucose as byproduct was found after 12 h of the reaction (Fig. 4B). Ma *et al*. 2006 [24] using permeabilized *P. putida* cells showed the maximum yield of 45%, which was lower than that obtained in this work. Their study used a permeabilizing process with the mixture of 2% (w/v) toluene and 0.2% (w/v) EDTA. However, the use of toluene is considered toxic for food application [25, 26].

### Scaling Up Trehalose Production in Bioreactor

**Fig. 5:**
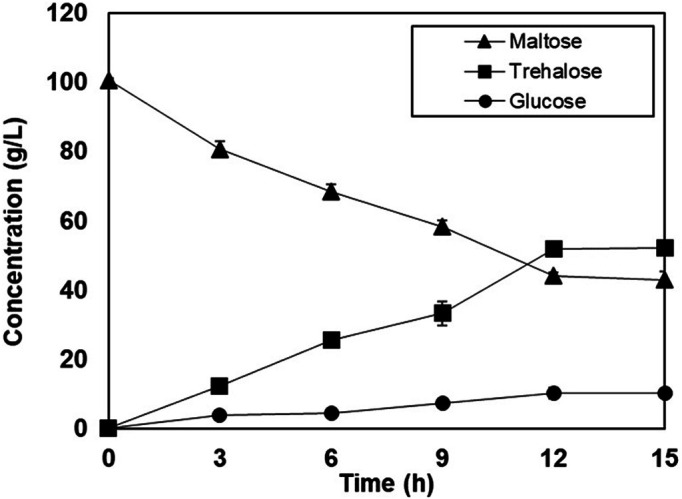
Trehalose production from maltose by permeabilized cells of *P. monteilii* TBRC 1196 in 1 L bioreactor. The reactions contained 100 g/l maltose in 50 mM potassium phosphate buffer (pH 8.0) and were incubated at 35°C for 15 h.

Trehalose conversion by the permeabilized cells was up-scaled in a 1 L bioreactor using 100 g/l maltose (purity>99%) as a substrate. The highest production of trehalose of 51.91 g/l was achieved by batch process after 12 h of incubation, while a glucose concentration of 10.33 g/l was observed as the byproduct. This was equivalent to the trehalose yield of 51.60% with 4.32 g/l/h productivity (Fig. 5A). Conversion of maltose to trehalose was usually performed by recombinant TreS expressed in heterologous bacterial hosts [4], due to low enzyme activity in the native microbes [11]. The trehalose yield in the range of 47-80% and formation of 0-21.7% glucose as the byproduct yield were obtained using isolated recombinant enzymes [6, 27-37]. In comparison with our process, the trehalose yield (51.60%) achieved from wild-type whole-cell catalysis in our study was lower than that obtained using some recombinant enzymes. However, our whole-cell catalytic process is considered advantageous in terms of simplicity as there is no need for the enzyme production and purification steps [20]. Compared to other works on trehalose production using permeabilized recombinant *E. coli* cells [11-12], the use of wild-type cells is advantageous as it requires no use of antibiotics and expensive inducer in the cell preparation step, although the trehalose yield is slightly lower (61.5-73.0 % by recombinant *E. coli*). The biocatalytic process using whole-cell *P. monteilii* TBRC 1196 developed in this study thus provides a promising alternative for trehalose production from maltose with potential application in bio-industry.

### Simultaneous Trehalose Purification and Ethanol Production

Among the 18 yeast strains that were screened on YNB-based media supplemented with glucose, maltose, or trehalose, only 2 strains were found to abstain from trehalose utilization. (Fig. S1). These two yeast strains were *S. cerevisiae* TBRC12153 and *S. cerevisiae* TBRC 3578. These strains converted glucose to ethanol with a concentration of 7.64 and 6.99 g/l, respectively, under the experimental conditions but could not produce ethanol from maltose (Fig. S2). *S. cerevisiae* TBRC12153 was selected for subsequent experiments as it showed higher ethanol concentration.

**Fig. 6:**
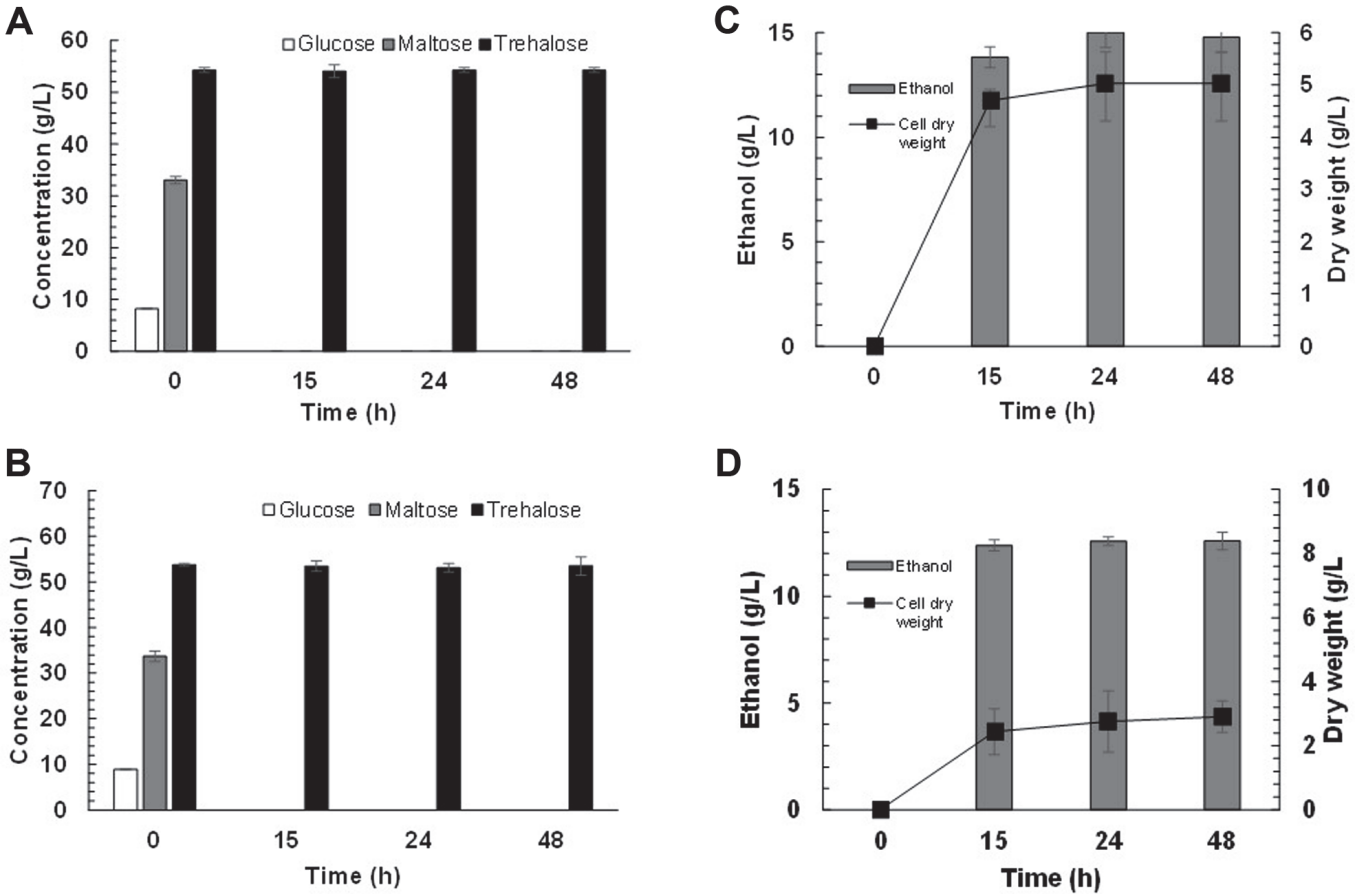
Sugar concentration from bioremoval of trehalose solution by *S. cerevisiae* TBRC 12153 with additional of yeast extract plus peptone (**A**) and ammonium sulfate plus mineral salts (**B**) and ethanol concentration in the bioremoval step by *S. cerevisiae* TBRC 12153 with additional of yeast extract plus peptone (**C**) and ammonium sulfate plus mineral salts (**D**). The reactions contained 54.78% trehalose, 33.66 g/l maltose and 8.87 g/l glucose and incubated at pH 6.5 and 30°C for 48 h.

For the bioremoval step, *S. cerevisiae* TBRC 12153 was incubated with the mixture from the trehalose production step containing 54.78% trehalose, 33.66 g/l maltose and 8.87 g/l glucose. The result showed that maltose and glucose can be converted to ethanol after 15 h without trehalose assimilation by using both nitrogen sources (Figs. 6A and 6B). This led to 0% and 53.45% of maltose and trehalose with 99.36% trehalose recovery. The ethanol concentration obtained using the processes supplemented with yeast extract plus peptone and ammonium sulfate plus mineral salts was 13.82 (Fig. 6C) and 12.37 g/l (Fig. 6D), respectively. However, the trehalose solution obtained from the process supplemented with inorganic salts is colorless compared with the yellowish solution from the process supplemented with organic nitrogen sources. This is considered advantageous in terms of cost in the downstream processing step before crystallization. The use of bioremoval is simple and specific compared to the application of physical or chemical methods such as adsorption or ion exchange, approaches which are limited by difficulty in separation of sugars with similar properties and molecular weights [38]. Our bioremoval process combining glucoamylase and yeast fermentation by SSF is considered cost effective compared to the conventional separation process. Moreover, yeast cells and ethanol can be recovered as co-products from the bioremoval process, making it a promising alternative for trehalose purification.

### Integrated Trehalose Production and Byproduct Bioremoval in Bioreactor

**Fig. 7:**
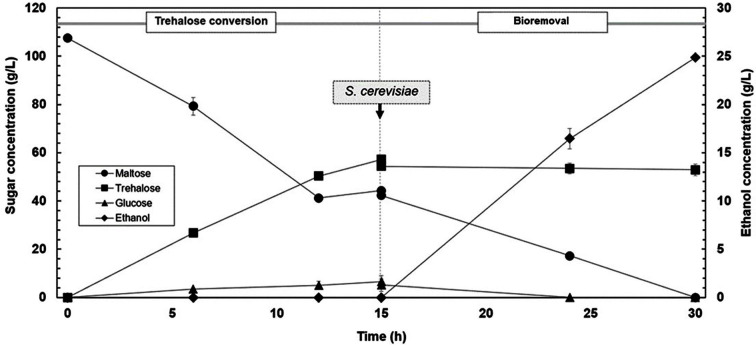
Time course of sugars conversion and ethanol production from one-pot process in 1 L bioreactor. Trehalose conversion was controlled at 35°C and pH 8.0 with an agitation at 100 rpm for 15 h. Bioremoval step was carried out at 30°C with an agitation at 200 rpm for 15 h.

A one-pot process integrating trehalose conversion and byproduct bioremoval in one bioreactor was demonstrated in this study. Maltose was converted to trehalose with a yield of 53.35% and a productivity of 3.55 g/l/h after 15 h (Fig. 7). The reaction contained 44.33 g/l of residual maltose and 6.49 g/l glucose as byproducts. Therefore, to separate trehalose from glucose and maltose in one step, the sugars were simultaneously converted to ethanol by addition of glucoamylase and *S. cerevisiae* TBRC12153. The residual glucose and maltose were converted to 24.9 g/l ethanol equivalent to a yield of 49.01% based on the total concentration of glucose and maltose after 15 h of fermentation. This process combines trehalose conversion and bioremoval steps in only one bioreactor, thus simplifying the trehalose production process from maltose and allowing recovery of high purity trehalose. Moreover, ethanol can be obtained as a co-product and can also be easily separated from trehalose by distillation. Application of the bioremoval step by *S. cerevisiae* has been reported in a few previous works on trehalose production with the use of isolated recombinant enzymes [38, 39]. Bioremoval by *S. cerevisiae* was also integrated with trehalose production by yeast cells displaying TreS on the cell surface, resulting in an ethanol yield of 40−45% after 24 h of fermentation. However, the trehalose solution obtained after the bioremoval step required decolorization with activated carbon due to the presence of yeast extract in the medium [40]. Compared to the previous reports, our work was the first to demonstrate integrated trehalose production by a wild-type strain with a bioremoval step by *S. cerevisiae*. Our developed process requires no enzyme isolation step and gives a colorless product solution with no need for the subsequent decolorization step. Thus it simplifies the overall production process for highly purified trehalose product.

In conclusion, a process of trehalose production by permeabilized cell of *P. montelii* TBRC 1196 was successfully demonstrated. The reaction conditions and permeabilization methods were optimized. Trehalose production was integrated with the byproduct bioremoval step using a selected strain of *S. cerevisiae*. This led to a final trehalose yield of 53.35% with 98.97% purity. The developed process is simple and efficient for application to trehalose production in bioindustry.

## Supplemental Materials

Supplementary data for this paper are available on-line only at http://jmb.or.kr.

## Figures and Tables

**Table 1:** Values by level for each factor in CCD.

Independent variable	Level				

	-1.414	-1	0	1	1.414
pH level	6.58579	7	8	9	9.41421
Temperature (°C)	20.8579	25	35	45	49.1421

**Table 2:** Effects of factors on trehalose synthase production by *P. monteilii* TBRC 1196.

Factors		Trehalose synthase activity (Unit/ml)
Temperature (°C)	25	148.38 ± 6.68^b^
	30	222.13 ± 11.54^c^
	35	371.80 ± 7.29^d^
	40	365.07 ± 7.49^d^
	45	88.72 ± 5.16^a^
Inoculum size (%)	5	187.04 ± 4.05^a^
	10	378.20 ± 3.85^d^
	15	350.96 ± 0.30^c^
	20	258.08 ± 8.30^b^
Shaking speed (rpm)	150	409.97 ± 6.58^b^
	200	442.62 ± 2.53^c^
	250	315.30 ± 11.24^a^
Maltose concentration (%, w/v)	0	115.29 ± 6.68^a^
	2.5	430.80 ± 6.89^b^
	5	472.19 ± 0.20^d^
	7.5	448.28 ± 4.86^c^
	10	437.18 ± 5.37^bc^

Each value is mean ± S.D. Means with different superscript letters are significantly different (*p* < 0.05, Duncan’s multiple range test).

**Table 3:** Experimental design of trehalose production by permeabilized cells.

Run	pH (A)	Temperature (B, °C)	Trehalose yield (%)
**1**	8	35	35.91 ± 0.52
**2**	7	25	21.36 ± 0.61
**3**	9.41	35	20.32 ± 0.23
**4**	8	20.86	8.39 ± 0.54
**5**	8	35	33.79 ± 1.23
**6**	8	49.14	5.36 ± 0.11
**7**	9	45	11.64 ± 0.37
**8**	8	35	37.16 ± 0.17
**9**	8	35	35.32 ± 0.06
**10**	8	35	34.38 ± 0.31
**11**	6.59	35	20.19 ± 0.05
**12**	7	45	10.04 ± 0.54
**13**	9	25	13.11 ± 0.24

**Table 4:** ANOVA for optimization of trehalose production; response: trehalose yield (%).

Source	Sum of Squares	df	Mean Square	F-value	*p*-value
**Model**	1668.67	5	333.73	105.02	< 0.0001	Significant
A-pH	5.21	1	5.21	1.64	0.2412	
B-Temp	36.44	1	36.44	11.47	0.0117	
AB	24.25	1	24.25	7.63	0.0280	
A²	382.04	1	382.04	120.22	< 0.0001	
B²	1383.09	1	1383.09	435.24	< 0.0001	
**Residual**	22.24	7	3.18			
Lack of Fit	15.31	3	5.10	2.94	0.1622	Not significant
Pure Error	6.94	4	1.73			
**Cor Total**	1690.91	12				

**Table 5:** The percentage error of predicted and observed experiment of trehalose production.

pH	Temperature (°C)	Trehalose yield (%)		Percentage error (%)

		Predicted	Observed	
8	35	35.31 ± 1.78	38.66 ± 0.54	8.66
7.5	35	33.86 ± 1.78	34.59 ± 0.32	2.11
8.5	35	33.06 ± 1.78	34.07 ± 0.29	2.97

**Table 6:** Trehalose production from various maltose concentrations by permeabilized *P. monteilii* TBRC 1196 cells.

Maltose (g/l)	Incubation time (h)	Trehalose (g/l)	Trehalose yield (%)
50	6	13.29 ± 1.04^a^	26.64 ± 2.08^c^
	12	22.73 ± 1.17^b^	45.58 ± 2.35^f^
	24	23.14 ± 0.59^c^	46.41 ± 1.19^g^
100	6	23.34 ± 0.95^d^	22.85 ± 0.93^b^
	12	52.90 ± 0.57^e^	51.79 ± 0.55^h^
	24	53.15 ± 1.48^f^	52.03 ± 1.45^i^
200	6	45.86 ± 0.21^g^	22.78 ± 0.11^a^
	12	86.90 ± 2.26^h^	43.16 ± 1.12^d^
	24	88.65 ± 0.78^i^	44.03 ± 0.39^e^

Each value is mean ± S.D. Means in same column with different superscript letters are significantly different (*p* < 0.05, Duncan’s multiple range test).
