# Supplementary material for: Integrated Whole-Cell Biocatalysis for Trehalose Production from Maltose Using Permeabilized Pseudomonas monteilii Cells and Bioremoval of Byproduct
Source: J Microbiol Biotechnol. 2022 May 20;32(8):1054–63. doi: 10.4014/jmb.2202.02028 (PMC9628947; doi:10.4014/jmb.2202.02028)
Supplement: Supplementary file 1 [file jmb-32-8-1054-supple.pdf]

J. Microbiol. Biotechnol.
https://doi.org/10.4014/jmb.2202.02028

Supplementary Materials

Integrated whole cell biocatalysis for trehalose production from maltose using permeabilized *Pseudomonas monteilii* cells and bioremoval of byproduct

Srisakul Trakarnpaiboon and Verawat Champreda*

Enzyme Technology Team, Biorefinery and Bioproduct Technology Research Group, National Center for Genetic Engineering and Biotechnology, 113 Thailand Science Park, Paholyothin RD., Klong Luang District, Pathumthani, 12120, Thailand

* Corresponding author. Tel.: +66 2564 6700 x 3446; fax: +66 2564 6707.

E-mail address: verawat@biotec.or.th (V. Champreda).

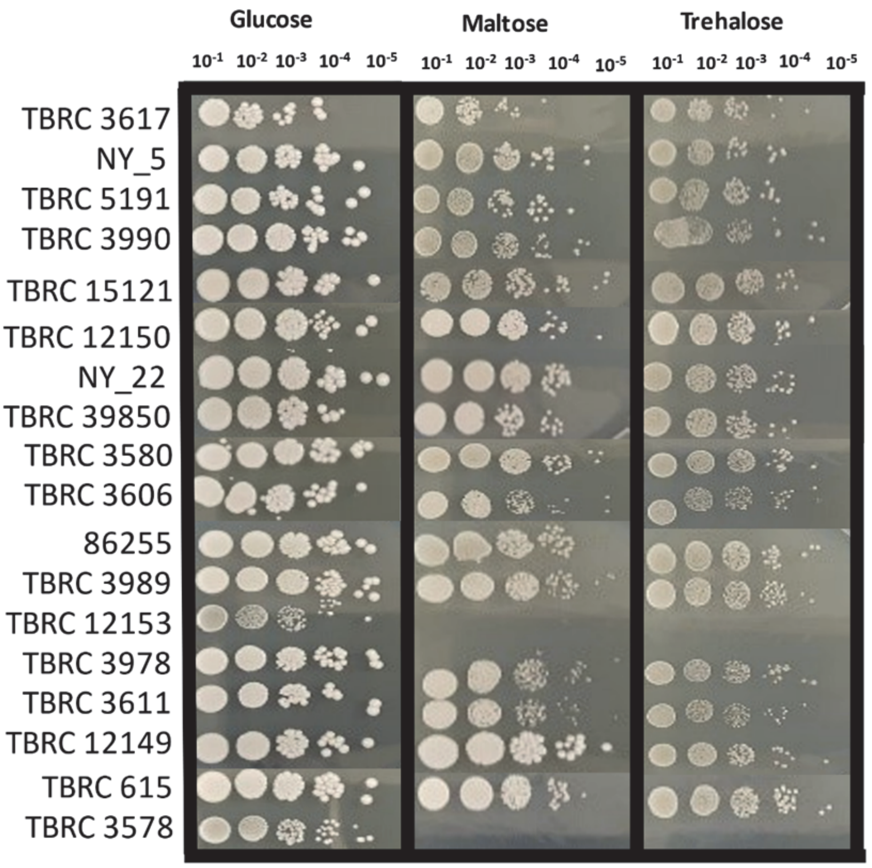

Fig.S1. Growth of yeast strains at 30 °C on YNB agar supplemented with 20 g/L of glucose, maltose, or trehalose for 48 h.

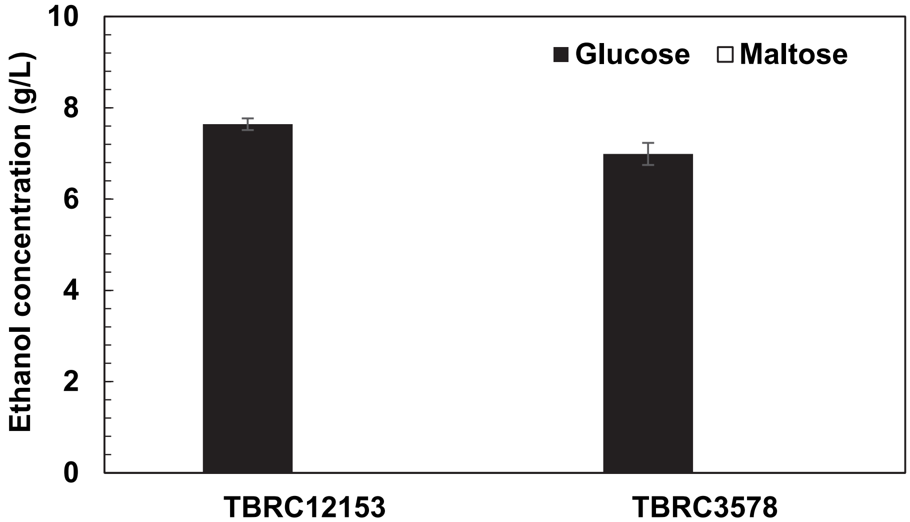

Fig.S2. Ethanol production by *S. cerevisiae* TBRC12153 and *S. cerevisiae* TBRC3578 in YNB broth containing 20 g/L glucose or maltose at 30 °C for 24 h.
